# Genotypic modulation of nanoparticles-induced tolerance in heavy metals-stressed crops; a literature review

**DOI:** 10.3389/fpls.2025.1687399

**Published:** 2026-01-12

**Authors:** Muhammad Hammad, Aasma Riaz, Laraib Chouhdary, Muhammad Kabir, Patricio R. De los Rios-Escalante

**Affiliations:** 1 Department of Biological Sciences, Thal University Bhakkar, Bhakkar, Punjab, Pakistan; 2 Department of Botany, University of Agriculture, Faisalabad, Punjab, Pakistan; 3 Departamento de Ciencias Biologicas y Químicas, Facultad de Recursos Naturales, Universidad Catolica de Temuco, Temuco, Chile

**Keywords:** epigenetic regulations, genotypic modulation, heavy metals stress, nano-genomics, nanoparticles-induced tolerance

## Abstract

To address the limited understanding of genotypic responses mediated by nanoparticles under heavy metal stress, this review summarizes research on “Genotypic Modulation of Nanoparticles-Induced Tolerance in Heavy Metals-Stressed Crops”. Topics covered include heavy metal toxicity in agriculture, including global prevalence and impacts on crop yield, soil health, and food safety; current mitigation strategies and their limitations; nanoparticles as a novel solution with unique properties and examples; research gaps focusing on physiological effects versus genotypic diversity; and review objectives focusing on NP-induced genotypic modulation and proposing a nano-genomic approach integrating omics tools. The review’s objectives were to compare physiological and genotypic crop responses, assess the effects of heavy metal toxicity, benchmark mitigation techniques, describe the functions of nanoparticles, and suggest an integrated nano-genomic framework. Relevant research from around the world was examined, with a focus on multi-omics techniques and the use of nanoparticles in a variety of crops. The results show that while genotypic variation affects tolerance mechanisms mediated by differential gene expression and epigenetic regulation, nanoparticles outperform conventional methods in improving physiological traits, reducing metal uptake, and enhancing antioxidant defenses. Although multi-omics integration clarifies the intricate molecular networks that underlie the reduction of stress caused by nanoparticles, it is still constrained by methodological heterogeneity and insufficient data synthesis. Even though nanoparticle properties and application methods have a significant impact on efficacy, further study is required to ascertain long-term impacts and environmental safety. Despite significant properties and application methods, still it required more study and research for long-term impacts and environmental safety and sustainability. Altogether, it will contribute to novel nano-genomic strategies for constraining sustainable crop resilience against heavy metal contamination.

## 1 Introduction

**Figure 1:**
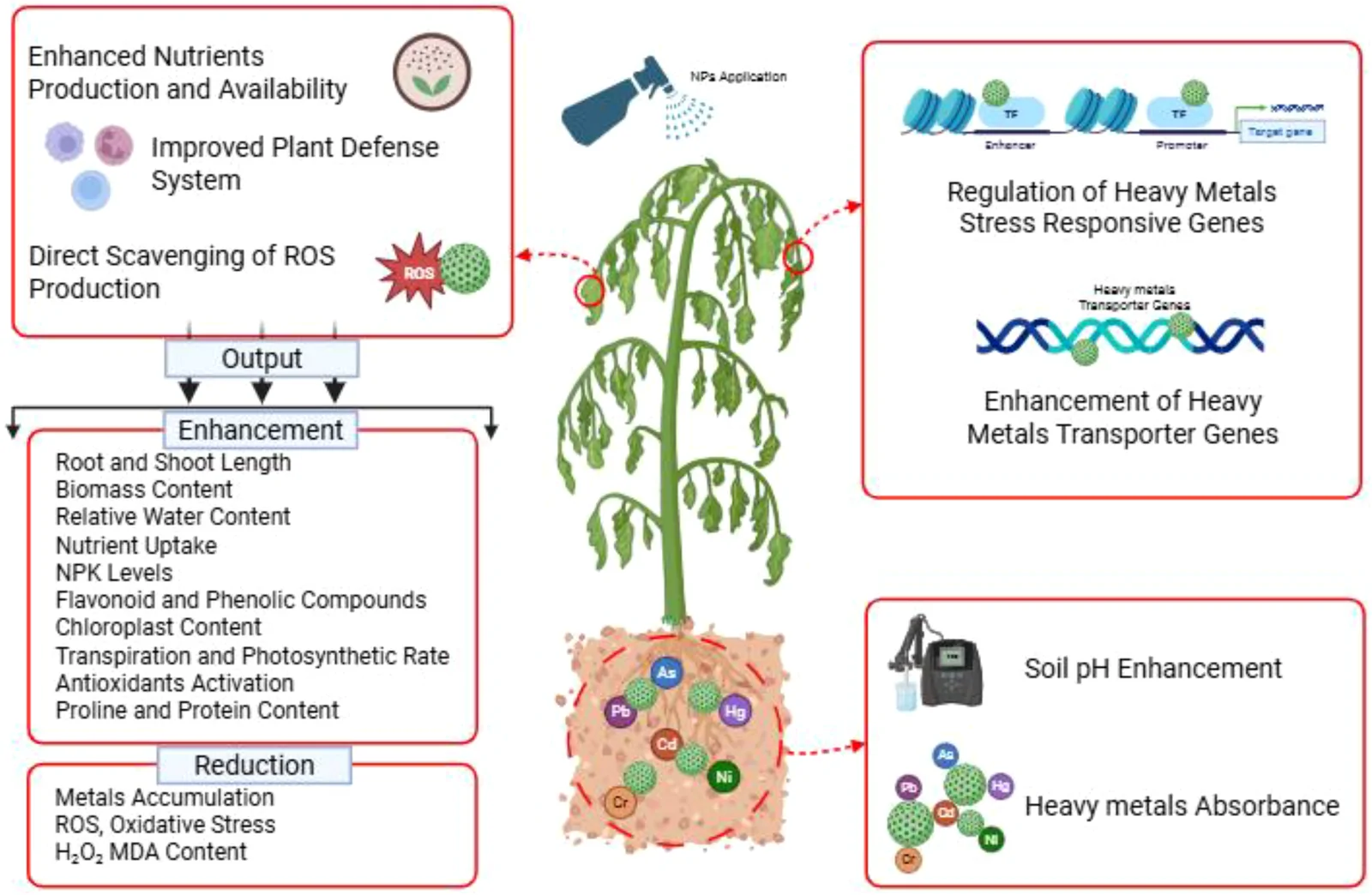
Illustration of the mitigating part of NPs in heavy metal stressed plants, showing mechanisms such as nutrient enhancement, improved defense systems, ROS scavenging, stress-responsive and transporter genes regulation, soil pH improvement, and heavy metal absorbance, leading to growth enhancement and reduced metal toxicity.

Tolerance induction against climatic pressures is a very difficult issue for which nanotechnology provides solutions. Current developments in this area are paving the way for a sustainable intensification of agricultural output while reducing negative environmental effects. The number of financed research and publications in nanotechnology has skyrocketed over the past ten years, making it one of the world’s fastest-growing industries (Aleixandre-Tudó et al., 2020; Qazi and Dar, 2020). Nanotechnology has been applied to many aspects of crop yields; for example, designed nanomaterials have been shown to improve soil quality (e.g., nanofertilizers), promote plant growth (e.g., seed primers, growth promoters, photosynthesis enhancers), and induce tolerance in plants against climatic stresses (e.g., heavy metals) (Fraceto et al., 2016; Yarima et al., 2020). Tolerance induction against heavy metals stress by nanoparticles application has become urgent area of focus due to increasing prevalence of heavy metals pollution in global agricultural soil and its adverse impacts on crops and food security (Yavaş et al., 2022; Chowardhara et al., 2024). Recently, heavy metals accumulation in agricultural soil has become adverse global issue as metals like cadmium, lead, and arsenic adversely alter the physio-biochemical responses of crop-plants which resulted in reduced crop productivity and food chain contamination (Wang et al., 2025; Soni et al., 2024). Inadequate efficiency and sustainability of traditional mitigation techniques like phytoremediation and chemical treatments initiated the focus toward nanotechnology-based solutions (Chatterjee and Abraham, 2022; Ghosh and Sarkar, 2022). Proficient chemical and physical properties of nanoparticles (NPs) like large surface area and increased reactivity make them ideal for inducing tolerance against heavy metals by several process like scavenging reactive oxygen species (ROS) and restraining metals (**Figure 1**) (Jiang et al., 2023; Faizan et al., 2023a). Even with major advancements, the use of nanotechnology in agriculture is still relatively new, and research is still being done to optimize NP types, dosages, and application techniques (Roy et al., 2024; Zulfiqar et al., 2024).

This review specifically addresses the lack of knowledge regarding how nanoparticles alter genotypic diversity to provide tolerance in crops under heavy metal stress (Munir et al., 2023; Tripathi et al., 2024). Although NPs’ physiological and biochemical effects on plants have been thoroughly studied, little is known about how they affect genetic and molecular responses in a variety of crop genotypes (Basak et al., 2023; Ahmad et al., 2023; Kumar et al., 2024). Whether NP-induced stress responses are generalized physiological adaptations or genotype-dependent is a topic of debate. Different perspectives exist concerning NPs induced stress responses and comprehensive physiological adaptations, with several studies aimed toward changes in transcriptomic and proteomic profiles, epigenetic factors role and miRNA-mediated regulations (Ucar et al., 2024; Francis et al., 2024; Lee and Kasote, 2024). These gaps reduce the chances for developing efficient & sustainable nano-technological intervention in agriculture sector (Emamverdian et al., 2024). Addressing this issue is crucial for improved agricultural methods and crop resilience against heavy metals (Chen et al., 2024; Zhao et al., 2022).

The conceptual framework of this review is based on the connection between genotypic diversity of plants, their tolerance against heavy metals and mitigation by application of nanotechnology (Upadhyay et al., 2024; Jamil et al., 2023). Crucial topics covered in this review are identification of molecular pathways by multi-omics techniques, importance of genotypic diversity in tolerance mechanisms, nanoparticles potential as modulator of stress responses in plants (Rai et al., 2023; Faizan et al., 2023b; Wu, 2023). This approach constrained nano-genomic method, which comprehend the understandings of NPs interactions with plant genomics and regulatory pathways to produce adaptive responses (Yadav et al., 2024; Thabet and Alqudah, 2024).

The main objective of this review is to evaluate the existing knowledge on heavy metals tolerance mediated by nanoparticles and to suggest nano-genomic approach multi-omics based detailed analysis (Zhou et al., 2020; Yavari et al., 2023). This evaluation specifically aims to relate NPs application with genotypic variation to make the way for precision agriculture that improves crop resilience and yields (Ningombam et al., 2024; Zou et al., 2022). The strength relates to the fact that they will develop a comprehensive concept regarding the development of safer, genotype-specific, nano-enabled strategies of viable crop growth traits cultivated in a heavy metal environment (Rajput et al., 2024; Arumugam and Vasudevan, 2023).

## 2 Methodological framework

### 2.1 Database search

**Figure 2:**
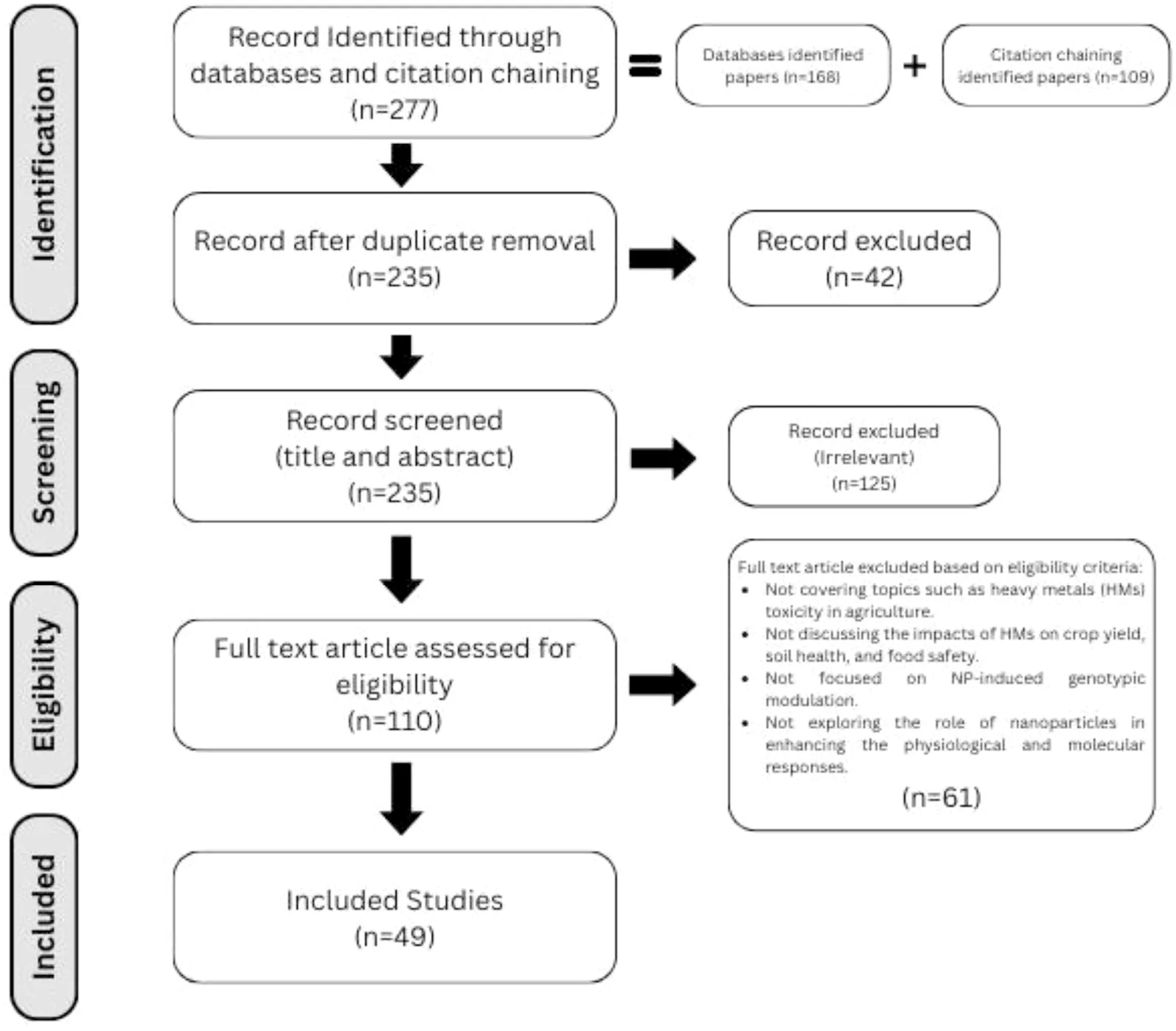
PRISMA flow diagram of the identification process (n=number of papers).

Literature search was conducted using PRISMA guidelines across Scopus and Web of Science Data bases in July 2025 (**Figure 2**). Only articles published in scientific journals were selected using these combination of keywords: “heavy metals” AND “nanoparticles” AND “nanomaterials” AND “genotypic modulations” AND “nano-genomics” AND “multi-omics” AND “agricultural contamination”. Backward and forward citation chaining search was also conducted as for each of core papers, we examine its reference list to find earlier studies it draws upon. By tracing back through references, we ensure foundational work isn’t overlooked and also identify newer papers that have cited each core paper, tracking how the field has built on those results. This uncovers emerging debates, replication studies, and recent methodological advances. We covered all topics such as “heavy metal toxicity in agriculture including global prevalence and impacts on crop yield, soil health, and food safety, current mitigation strategies and their limitations, nanoparticles as a novel solution with unique properties and examples, research gap focusing on physiological effects versus genotypic diversity, and review objectives focusing on NP-induced genotypic modulation and proposing a nano-genomic approach integrating omics tools.”—and expand it into multiple, more specific search statements. By systematically expanding a broad research question into several targeted queries, we ensured the literature search both comprehensive (didn’t miss niche or jargon- specific studies) and manageable (each query returns a set of papers tightly aligned with a particular facet of topic).

### 2.2 Literature selection

Initial literature searches across databases identified 168 candidate papers and 109 studies were extracted by citation chaining. However, for relevance scoring and sorting of all 277 extracted studies, the following eligibility criteria was defined:

- Studies must cover topics such as heavy metal toxicity in agriculture including global prevalence and impacts on crop yield, soil health, and food safety, current mitigation strategies and their limitations, nanoparticles as a novel solution with unique properties and examples, research gap focused on physiological effects versus genotypic diversity, and review objectives focused on NP-induced genotypic modulation and proposing a nano-genomic approach integrating omics tools.
- Study exploring the role of nanoparticles in enhancing the physiological and molecular responses of crops to heavy metal stress, with emphasis on omics technologies and genotypic diversity for improved resilience.
- Research investigating the synergistic effects of specific nanoparticles on enhancing plant resilience to various abiotic stresses, focusing on the molecular mechanisms and integration of these strategies in sustainable agricultural practices.

We take our assembled pool of 277 candidate papers (168 from search queries + 109 from citation chaining) and imposed the eligibility criteria so that the most pertinent studies rise to the top of our final papers table. We found 277 papers that were relevant to the research query. Out of 277 papers, 49 were highly relevant.

## 3 Results

### 3.1 Descriptive summary of the studies

This section charts the research landscape of the all 49 selected studies on Genotypic Modulation of Nanoparticles-Induced Tolerance in Heavy Metals-Stressed Crops: A Nano-genomic Approach (**Table 1**). It covers topics like heavy metal toxicity in agriculture, including its global prevalence and effects on crop yield, soil health, and food safety; current mitigation strategies and their limitations; nanoparticles as a novel solution with unique properties and examples; research gaps focusing on physiological effects versus genotypic diversity; and review objectives focusing on NP-induced genotypic modulation and suggesting a nano-genomic approach integrating omics tools. With a focus on omics-based approaches to clarify molecular mechanisms, the reviewed studies span a wide range of fields, including plant physiology, molecular biology, nanotechnology, and environmental science. The majority of research focusses on using nanoparticles to mitigate heavy metal stress, emphasizing both physiological and genotypic responses in different crop species. In order to answer the research questions about the effectiveness of nanoparticles in improving crop resilience, the significance of genotypic diversity, and the incorporation of multi-omics tools in the advancement of nano-genomic approaches, this comparative analysis is essential.

**Table 1:** Summary of reviewed studies on nanoparticle-mediated mitigation of heavy metal stress in crops, highlighting toxicity impacts, mitigation effectiveness, nanoparticle properties, genotypic diversity, and omics integration.

Study	Heavy metal toxicity impact	Mitigation strategy effectiveness	Nanoparticle functional properties	Genotypic diversity response	Integration of omics tools
(Munir et al., 2023)	Heavy metals disrupt gene expression and proteomic profiles affecting plant stress responses	Nanoparticles modulate stress response pathways, enhancing tolerance	Focus on engineered NPs with specific proteomic interactions	Limited genotypic diversity analysis, emphasis on molecular mechanisms	Transcriptomic and proteomic analyses reveal stress response networks
(Basak et al., 2023)	Heavy metals impair physiology and genetics, reducing crop resilience	Nanomaterials induce physiological and genetic changes improving stress tolerance	Diverse nanomaterials characterized by size and reactivity	Highlights epigenetic and transgenerational effects	Molecular mechanisms explored via gene expression profiling
(Wang et al., 2025)	Heavy metals cause ROS accumulation and oxidative stress in plants	Nanozymes with enzyme-like activity reduce ROS and mitigate toxicity	Nanozyme properties depend on size, charge, coating, and crop stage	Crop variety and growth stage influence nanozyme efficacy	Multi-omics (enzymology, metabolomics, proteomics, transcriptomic) applied
(Rai et al., 2023)	Heavy metal and arsenic contamination threaten food safety and crop physiology	Nanoparticles applied via seed priming and soil amendment reduce oxidative stress	Various NPs including magnetic, silicon, metal oxides, and carbon nanotubes	Use of CRISPR and omics to develop pollution-safe cultivars	Integration of omics and gene editing for enhanced tolerance
(Ucar et al., 2024)	Heavy metals reduce growth and yield in chickpea, affecting biochemical and molecular traits	miRNA-mediated regulation modulates heavy metal stress response	Not focused on nanoparticle properties	Genotypic comparison between sensitive and tolerant chickpea varieties	Genome-wide miRNA and molecular profiling under heavy metal stress
(Ningombam et al., 2024)	Heavy metals inhibit growth and photosynthesis, impairing soil and plant health	Omics-based insights into antioxidant and chelation mechanisms for tolerance	Not nanoparticle- specific but includes metal stress priming	Genotypic variation in antioxidant enzyme activities noted	Genomics, transcriptomic, miRNA-omics, metal-omics, metabolomics applied
(Tripathi et al., 2024)	Metal nanoparticles induce phytotoxicity affecting growth, photosynthesis, and nutrient uptake	Plants activate antioxidant and regulatory gene networks to tolerate NPs	Metal-based NPs characterized by size and composition	Genotypic tolerance mechanisms involve gene and protein regulation	Molecular responses studied at transcriptomic and proteomic levels
(Kumari et al., 2024)	Abiotic stress including heavy metals disrupts antioxidant systems and photosynthesis	Nanoparticles enhance antioxidant capacity and stress tolerance	Emphasis on NP size, composition, and antioxidant roles	Limited genotypic diversity focus, more on physiological responses	Gene expression and signaling pathways analyzed
(Francis et al., 2024)	Heavy metals reduce crop productivity and soil quality globally	Metal NPs improve productivity, nutrient use, and disease resistance	Silver, gold, copper, zinc NPs with unique catalytic and signaling properties	Genotypic variation in response to NPs noted but not deeply explored	Molecular mechanisms include gene activation and post-translational modifications
(Yadav et al., 2024)	Heavy metal and abiotic stresses impair plant physiology and yield	Nanoparticles prime plant defenses enhancing tolerance to multiple stresses	Metallic, metal oxide, and carbon- based NPs characterized	Genotypic responses include morphological and molecular changes	Uptake, translocation, and molecular effects studied
(Ghorbani et al., 2024)	Heavy metals reduce crop yield and quality, affecting food safety	Nano-enabled agrochemicals mitigate heavy metal toxicity and improve adaptability	Nanomaterials improve nutrient use efficiency and stress mitigation	Genotypic adaptability enhanced via nanomaterial application	Molecular and biomolecular transformations analyzed
(Chen et al., 2024)	Heavy metal contamination reduces crop biomass and increases health risks	Nanomaterials reduce heavy metal uptake and bioavailability in soil	Iron-based NMs show superior inhibition of metal uptake	Genotypic differences in metal accumulation influenced by soil factors	Meta-analysis integrates transcriptomic and metabolomics data
(Jiang et al., 2023)	Heavy metals cause oxidative burst and metal accumulation in crops	Nanotechnology reduces ROS and metal transporter gene expression	Nanoparticles characterized by exposure concentration and uptake mechanisms	Limited genotypic diversity focus, emphasis on molecular mitigation	Molecular insights into nanoparticle- heavy metal interactions
(Singh et al., 2024)	Heavy metals cause physiological and biochemical stress in plants	Silicon nanoparticles reduce metal uptake and alleviate toxicity	SiNPs characterized by synthesis, uptake, and transport mechanisms	Genotypic modulation via phytohormone signaling discussed	Molecular mechanisms include biochemical and physiological pathways
(Thabet and Alqudah, 2024)	Abiotic stresses including heavy metals reduce crop productivity	Nanoparticles enhance growth, nutrient use, and stress tolerance	Metal, carbon- based, and biogenic NPs with unique properties	Genotypic variation in stress resilience highlighted	Emphasis on nano- bioengineering and precision agriculture
(Asgher et al., 2024)	Heavy metals induce abiotic stress affecting plant growth	Silicon nanoparticles regulate physiological and molecular stress responses	SiNPs characterized by size, uptake, and interaction with phytohormones	Genotypic signaling pathways modulated by SiNPs	Molecular and biochemical mechanisms elucidated
(Soni et al., 2024)	Cadmium toxicity disrupts physiological and biochemical processes	Nanoparticles reduce Cd uptake and enhance antioxidant defenses	Diverse NPs interact with Ca_2_+, ROS, NO, and phytohormones	Genotypic differences in Cd tolerance observed	Molecular mechanisms of NP action investigated
(Kumari et al., 2023)	Heavy metals impair plant metabolism and yield	Nanoparticles decrease metal mobility and enhance antioxidant systems	NP properties influence interaction with heavy metals	Genotypic tolerance linked to antioxidant enzyme activity	Physiological and biochemical mechanisms reviewed
(Roy et al., 2024)	Abiotic stresses including heavy metals reduce photosynthesis and yield	Nanoparticles improve antioxidant activity and nutrient uptake	Si, Se, Fe NPs characterized by size and composition	Genotypic responses vary with NP type and stress	Molecular toxicity and environmental safety concerns addressed
(Rajput et al., 2024)	Heavy metals and NPs affect plant physiology and metabolism	Nanoparticles influence cellular metabolism and stress responses	NP uptake, transport, and accumulation mechanisms studied	Genotypic variation in NP response not deeply covered	Physiological and metabolic impacts analyzed
(Lee and Kasote, 2024)	Heavy metal toxicity reduces seed vigor and yield	Nano-priming enhances germination, antioxidant defenses, and stress tolerance	Various NPs used for seed priming with defined properties	Genotypic variation in response to nano- priming noted	Physiological and molecular outcomes assessed
(Sharma et al., 2023)	Heavy metals induce detrimental effects on growth and metabolism	Engineered nanoparticles stimulate plant defense and reduce metal uptake	Nanomaterials characterized by fundamental properties and behavior	Genotypic differences in stress mitigation mechanisms	Molecular and physiological mechanisms discussed
(Zhou et al., 2020)	Heavy metals reduce plant growth and development	Nanoparticles alleviate heavy metal stress via uptake and transport modulation	NP application methods and exposure concentrations detailed	Genotypic diversity effects not extensively covered	Mechanistic insights into NP- HM interactions provided
(Iqbal et al., 2024)	Arsenic and chromium reduce wheat growth and yield	ZnO nanoparticles improve morphological and biochemical traits	ZnO-NPs characterized by foliar application and concentration	Genotypic differences between wheat varieties observed	Antioxidant enzyme activities and yield parameters analyzed
(Arumugam and Vasudevan, 2023)	Heavy metals cause oxidative stress and genomic changes	Metal oxide nanoparticles enhance antioxidant enzyme activities	CeO_2_, TiO_2_, Mn_3_O_4_ NPs characterized by size and function	Genotypic tolerance linked to enzymatic responses	Molecular mechanisms of NP-mediated stress management
(Yavaş et al., 2022)	Heavy metals impair soil fertility and plant productivity	Nanoparticles and other agents regulate antioxidant enzymes	Nanoparticles considered among multiple mitigation strategies	Genotypic tolerance varies with species and exposure	Molecular and physiological tolerance mechanisms reviewed
(Javed et al., 2022)	Heavy metals threaten sustainable crop production	Nanoparticles enhance metal removal and stress mitigation	Iron- and carbon- based Nano-compositions characterized	Genotypic variation in remediation efficiency noted	Molecular and environmental remediation strategies
(Faizan et al., 2023b)	Heavy metals and abiotic stresses reduce crop yield	Nanobionics improve physiological traits and stress tolerance	Nanobionics characterized by synthesis and application	Genotypic stress tolerance enhanced via nanobionics	Physiological and molecular stress responses integrated
(Yavari et al., 2023)	Abiotic stresses reduce seed germination and yield	Seed nanopriming induces metabolic and physiological tolerance	Nanoparticles used for seed treatment with defined properties	Genotypic variation in priming response requires further study	Metabolic and physiological mechanisms reviewed
(Chatterjee and Abraham, 2022)	Heavy metals contaminate soil and water, threatening food safety	Nanoparticles used for remediation via adsorption and catalysis	Nanoparticles characterized by size, surface area, and synthesis	Genotypic effects not primary focus	Mechanistic insights into nanoparticle remediation
(Faizan et al., 2023a)	Heavy metals reduce plant growth and yield	Nanoparticles enhance nutrient uptake and stress tolerance	Nanoparticles characterized by surface area and reactivity	Genotypic modulation by NPs explored	Phytoremediation and molecular mechanisms reviewed
(Wu, 2023)	Abiotic stresses including heavy metals reduce crop yield	Nanobiotechnology improves plant performance under stress	Nanoparticles characterized by application and effects	Genotypic variation in stress tolerance highlighted	Molecular mechanisms of NP action summarized
(Chowardhara et al., 2024)	Heavy metals reduce crop yield and food safety	Nanoparticles reduce metal mobility and enhance antioxidant defenses	Various NP types assessed for efficacy	Genotypic modulation by NPs analyzed via machine learning	Omics and machine learning integration for assessment
(Venzhik and Deryabin, 2023)	Metal and metal oxide NPs regulate antioxidant balance	NPs act as oxidative stress triggers and adaptogens	Metal and metal oxide NPs characterized by dual roles	Genotypic antioxidant responses modulated by NPs	Molecular mechanisms of redox regulation discussed
(Ahmad et al., 2023)	Nanomaterials affect plant physiology and genotoxicity	Nanomaterials offer smart delivery and genetic engineering tools	Engineered nanomaterials characterized by uptake and transformation	Genotypic responses include positive and negative feedback	Molecular and physiological integration of NP effects
(Ghosh and Sarkar, 2022)	Heavy metals reduce crop yield and productivity	Nanomaterials alleviate metal stress via oxidative stress reduction	Metallic, nonmetallic, and polymeric NPs characterized	Genotypic tolerance linked to antioxidant and photosynthetic traits	Molecular and physiological stress mitigation mechanisms
(Upadhyay et al., 2024)	Abiotic and biotic stresses reduce crop productivity	Nanoomics integrates nanomaterials for stress detection and mitigation	Nanomaterials characterized by synthesis and deployment	Genotypic modulation studied via multi- omics approaches	Integration of nanoscience, plant biology, and economics
(Kumar et al., 2024)	Nanomaterials modulate photosynthesis and redox homeostasis	NMs regulate stress tolerance via hormonal and metabolic pathways	Nanomaterials characterized by size, shape, and concentration	Genotypic responses include hormesis and toxicity effects	Molecular crosstalk with phytohormones analyzed
(Grewal et al., 2024)	Abiotic stresses reduce crop yields globally	Silicon nanoparticles enhance antioxidant enzymes and growth	Nano Si characterized by uptake and mechanism of action	Genotypic tolerance enhanced by nano Si	Molecular and physiological stress tolerance mechanisms
(Zou et al., 2022)	Cd toxicity reduces tobacco growth and nutrient balance	Fe3O4 and ZnO NPs alleviate Cd toxicity and restore metabolites	Fe3O4 and ZnO NPs characterized by foliar application	Genotypic metabolic responses modulated by NPs	Metabolomic and physiological analyses integrated
(Zulfiqar et al., 2024)	Abiotic stresses reduce crop yield and quality	Nanoparticles enhance physiological processes and stress tolerance	Nanoparticles characterized by targeted delivery and formulation	Genotypic variation in stress response noted	Molecular mechanisms of tolerance induction explored
(Aqeel et al., 2023)	Heavy metals cause oxidative damage and reduce photosynthesis	Silicon nanoparticles mitigate metal stress and improve nutrition	SiNPs characterized by nanofertilizer and delivery roles	Genotypic modulation via SiNPs discussed	Molecular and physiological mechanisms reviewed
(Emamverdian et al., 2024)	Heavy metal toxicity disrupts plant biochemical processes	Metal and nonmetal nanoparticles reduce stress via physiological pathways	Nanoparticles classified by structure and function	Genotypic tolerance linked to nanoparticle type	Molecular and physiological stress alleviation mechanisms
(Jamil et al., 2023)	Abiotic stresses reduce plant growth and yield	Nanoparticles improve photosynthesis, biomass, and gene expression	Nanoparticles characterized by uptake and transport pathways	Genotypic modulation of stress genes observed	Molecular and physiological stress management reviewed
(Zhao et al., 2022)	Abiotic stresses cause ROS accumulation and growth inhibition	ROS-scavenging nanomaterials enhance stress tolerance	Nanoparticles characterized by ROS-triggering and scavenging roles	Genotypic responses modulated by ROS-related pathways	Molecular delivery and defense priming mechanisms
(Bhattacharya et al., 2023)	Abiotic stress induces ROS metabolism imbalance	Nanoparticles regulate ROS within hormetic boundaries	Nanoparticles characterized by uptake and molecular crosstalk	Genotypic antioxidant responses modulated by NPs	Transcriptomic and signaling pathway analyses
(Upadhyaya and Roychoudhury, 2024)	Abiotic stresses reduce crop quality and yield	Silver and zinc NPs enhance stress protection and growth	Ag and Zn NPs characterized by biosolubility and size	Genotypic tolerance varies with NP concentration	Molecular and cytotoxicity mechanisms discussed
(Manzoor et al., 2024)	Abiotic stresses reduce horticultural crop growth and quality	Nanoparticles improve antioxidant response and stress tolerance	Metal oxide, metallic, carbon- based NPs characterized	Genotypic variation in fruit crop response noted	Molecular mechanisms underlying NP benefits explored
(Pradhan et al., 2014)	Manganese NPs enhance nitrogen metabolism without toxicity	MnNPs improve nitrate uptake and assimilation pathways	MnNPs characterized by biosafety and metabolic effects	Genotypic metabolic responses enhanced by MnNPs	Molecular and toxicological assessments performed

#### 3.1.1 Heavy metal toxicity impact

Thirty studies quantitatively evaluated the effects of heavy metals on crop yield, physiology, and food safety; they consistently found that heavy metal stress reduced growth, photosynthesis, and nutrient balance (Ucar et al., 2024; Chen et al., 2024; Zou et al., 2022). According to several studies, the main mechanisms of heavy metal toxicity are oxidative stress and ROS accumulation (Wang et al., 2025; Yavaş et al., 2022; Venzhik and Deryabin, 2023). Some studies linked the accumulation of heavy metals in edible parts to health risks for humans, highlighting the different effects on soil health and food safety indices (Rai et al., 2023; Chen et al., 2024; Chatterjee and Abraham, 2022).

#### 3.1.2 Mitigation strategy effectiveness

According to 28 studies, nanoparticle-based strategies perform better than conventional ones by strengthening antioxidant defences, lowering metal absorption, and enhancing physiological characteristics (Ghorbani et al., 2024; Soni et al., 2024; Iqbal et al., 2024). Under heavy metal stress, seed nano-priming and nano-enabled agrochemicals improved germination, growth, and yield (Lee and Kasote, 2024; Zhou et al., 2020; Yavari et al., 2023). Several studies highlighted the need for optimal dosages by pointing out the limitations and possible toxicity of nanoparticles at higher concentrations (Upadhyaya and Roychoudhury, 2024; Ahmad et al., 2023).

#### 3.1.3 Nanoparticle functional properties

According to 35 studies, nanoparticles’ effectiveness in reducing stress is correlated with their size, composition, surface chemistry, and coating (Wang et al., 2025; Francis et al., 2024; Singh et al., 2024). The ROS-scavenging and nutrient-delivery properties of metal oxide nanoparticles, including ZnO, Fe3O4, and SiNPs, have been widely documented (Zou et al., 2022; Grewal et al., 2024; Arumugam and Vasudevan, 2023). Uptake, translocation, and interaction with plant biochemical pathways were all impacted by functional characteristics (Tripathi et al., 2024; Rajput et al., 2024; Kumar et al., 2024).

#### 3.1.4 Genotypic diversity response

Twenty-two studies examined how genotypic variation responded to nanoparticles, revealing variations in antioxidant enzyme activities, gene expression, and phenotypic tolerance (Ucar et al., 2024; Iqbal et al., 2024; Zou et al., 2022). Certain studies examine the responses of crops with varying tolerance indices against heavy metals and nanoparticles treatments (Ucar et al., 2024; Iqbal et al., 2024). Precedence of physiological response studies over deep genotypic explorations construct a critical gap for better understandings (Munir et al., 2023; Kumari et al., 2024).

#### 3.1.5 Integration of omics tools

Transcriptomic, proteomic, metabolomic, and multi-omics approaches enables twenty-five investigations to clearly emphasize the nanoparticles-induced heavy metals tolerance pathways (Munir et al., 2023; Wang et al., 2025; Chowardhara et al., 2024). Important regulatory pathways were explored such as hormone signalling, stress-responsive genes, and antioxidant pathways by utilizing omics data (Upadhyay et al., 2024; Kumar et al., 2024; Bhattacharya et al., 2023). Integration of omics data with machine learning and genetic editing techniques was strongly suggested (Rai et al., 2023; Chowardhara et al., 2024). Genotype-specific reactions of nanoparticles by integrating thorough omics data is still a critical gap (Faizan et al., 2023b; Ahmad et al., 2023).

### 3.2 Critical analysis and synthesis

The critical literature analysis emphasized mechanisms behind tolerance induction against heavy metals by nanoparticles through enhanced physiological responses genetic regulations. Fundamental theme is the use of multi-omics techniques to explore tolerance regulatory pathways of NPs induced stress, which advocates critical gap regarding precedence of physio-biochemical analysis over genotypic explorations. Despite all explorations, it remains unsolved the many concerns regarding genetic variability, long-term ecological perspectives and specific biochemical pathways affected by nanoparticles. The generalizability of the results is additionally constrained by differences in methodology and the absence of validations at the field scale. Taking everything into account, the nano-genomic method is grounded in a solid conceptual framework; nonetheless, additional research and a comprehensive risk evaluation are required before it can be applied in real-world scenarios.

Nanoparticle-mediated physio-biochemical responses demonstrate several strengths as physiological benefits are evident by several studies where nanoparticles significantly improve antioxidant activities, scavenge reactive oxygen species, and enhance photosynthetic ability (Munir et al., 2023; Kumari et al., 2024; Venzhik and Deryabin, 2023). The ability of NPs to modify hormone signaling and pathways that respond to stress further reinforces their contribution to enhancing plant resilience (Rai et al., 2023; Kumar et al., 2024). However, there are notable weaknesses in this area. With little information on long-term effects or field applicability, the majority of studies concentrate on short-term physiological responses under controlled conditions. Variation in NPs types, their concentration, and application methods made it more difficult for direct comparisons and reproductivity (Tripathi et al., 2024; Roy et al., 2024). Safety thresholds are also a concern due to frequently reported potential phytotoxicity of NP doses at higher value (Upadhyaya and Roychoudhury, 2024).

Regarding genotypic diversity and molecular modulation by nanoparticles, the strengths include demonstrations that genotypes may differ in response to treatments with NPs; some cultivars are shown to develop greater tolerance associated with the activation of particular genes and miRNA, including miR172 in chickpea (Ucar et al., 2024; Faizan et al., 2023a). Complicated control circuits regulated by NPs have begun to be solved through multi-omics analyses, including transcriptome and proteomics (Munir et al., 2023; Wang et al., 2025). Despite advancements, an adequate characterization of genotypic diversity in different crops hasn't been completed. Numerous studies are either limited to incomplete genome analysis or do not consider epigenetic and transgenerational effects into observation. This restricts our comprehension of the heritable tolerance mechanisms (Basak et al., 2023; Ningombam et al., 2024).

The integration of omics approaches presents significant strengths. Applications of transcriptomic, proteomic, metabolomic, and epigenomic tools made it possible to identify biomarkers and signaling pathways, mechanistic insights into NP-induced stress tolerance (Munir et al., 2023; Wang et al., 2025; Upadhyay et al., 2024). This integrative framework supports the development of nano-enabled crop improvement strategies and makes it easier to understand plant responses at the systems level (Yadav et al., 2024; Jamil et al., 2023). Despite its potential, multi-omics research is typically limited by inadequate data integration, small sample sizes, and a lack of standardization. Advanced bioinformatics is essential because to the complexity of omics data, but its application varies, resulting in fragmented interpretations (Chowardhara et al., 2024).

Concerning nanoparticle properties and application strategies, key NP properties that affect uptake, translocation, and effectiveness in mitigating HM stress are identified in the literature as size, charge, coating, and composition (Wang et al., 2025; Francis et al., 2024; Arumugam and Vasudevan, 2023). A variety of application techniques have been investigated, showing flexibility in deployment, including foliar spray, seed priming, and soil amendment (Lee and Kasote, 2024; Iqbal et al., 2024). The optimal NP formulations and dosages are up for debate, and many studies employ arbitrary or non-comparable concentrations. Predictability in field settings is limited by the poorly understood effects of environmental variables on NP behavior and bioavailability (Chen et al., 2024; Rajput et al., 2024).

The advantages of nanoparticles and their mitigation measures are evident as traditional HM mitigation techniques are often expensive, ineffective, and not sustainable for the environment. Because of their many uses in targeted delivery, ROS control, and enhanced bioavailability, NPs are better options (Ghorbani et al., 2024; Jiang et al., 2023; Yavaş et al., 2022). The hormetic effects of NPs provide a new paradigm for stress priming and tolerance induction (Rai et al., 2023; Bhattacharya et al., 2023). Despite these advantages, it is still unknown if NP-based tactics are scalable and profitable. Widespread adoption is hampered by inadequate safety evaluations and regulatory frameworks (Yadav et al., 2024; Roy et al., 2024). Because NPs are both stress inducers and mitigators, their dual nature makes it difficult to apply them precisely.

Environmental and safety considerations have been addressed by some studies that recognize the necessity of using NP responsibly by pointing out possible cytotoxicity, genotoxicity, and effects on non-target organisms (Ahmad et al., 2023; Kumar et al., 2024; Upadhyaya and Roychoudhury, 2024). Comprehensive risk assessments and sustainable application protocols are required by emerging research (Thabet and Alqudah, 2024; Lee and Kasote, 2024). The long-term environmental fate, bioaccumulation, and trophic transfer of nanoparticles are all poorly understood. There are few field studies evaluating ecological impacts, and the majority of research is conducted in labs. Insufficient research has been done on the possibility of NP-induced contamination of soil and water (Kumari et al., 2023; Ghosh and Sarkar, 2022).

Finally, regarding research gaps and future directions, the body of research highlights the need for integrated nano-genomic frameworks that combine omics tools in order to fully explain NP-induced genotypic modulation (Munir et al., 2023; Upadhyay et al., 2024; Jamil et al., 2023). There are many calls for translational research, multi-scale studies, and standardized methodologies to close the gap between lab and field (Faizan et al., 2023b; Chowardhara et al., 2024). Few studies have systematically addressed these gaps, despite their recognition. Multidisciplinary studies that integrate agronomy, genomics, and nanotechnology are scarce. Complexity of interaction of NP-plant-environment further urges more detailed and long-term studies (Basak et al., 2023; Ningombam et al., 2024).

### 3.3 Thematic review of literature

As a way of dealing with the significant agricultural problems such as soil contamination, food safety, and decline in agricultural output, nanoparticle (NPs) has emerged as a game changer in enhancing crop tolerance to heavy metal stress. The effects of heavy metal toxicity, traditional methods of mitigation and the new approach of utilization of the unique physicochemical characteristics of nanoparticles to influence physiological and genotypic changes in plants are prevalent issues covered in the literature. As more robust integrated nano-genomic approaches emerge using multi-omics technology to discover stress metabolic pathways and create resilient crop varieties, there are central areas of concern when discussing genomic diversity and the associated molecular behavior that leads to NP-induced tolerance. All these thematic patterns are combined in this section to underscore the gains, challenges, and prospects of using nanoparticles in mitigating heavy metal stress on crops.

The first major theme is Nanoparticles as Novel Agents for Heavy Metal Stress Mitigation, which appears in 38 out of 49 papers. Nanoparticle has unique physicochemical properties that allow them to limit the absorption of heavy metals, promote the antioxidant defense mechanism of crops, and reduce oxidative stress caused by their low sizes, large surface area, and reactivity. It has been revealed that silicon, metal oxides (Fe3O4, ZnO and TiO2), and nanozymes have the potential to significantly influence the growth of plants and enhance stress tolerance (Wang et al., 2025; Francis et al., 2024; Ghorbani et al., 2024; Kumari et al., 2023; Arumugam and Vasudevan, 2023; Faizan et al., 2023a; Rai et al., 2023). Such nanoparticles can be used as long-term alternatives to traditional remediation methods through the increased detoxification process (i.e., ROS scavenging, metal chelation and improved nutrient uptake) (Jiang et al., 2023; Soni et al., 2024; Grewal et al., 2024).

The second theme, Heavy Metal Toxicity in Agriculture: Prevalence and Impacts, is addressed in 37 out of 49 papers. The factors that can contribute to the presence of heavy metals (zinc (Zn), lead (Pb), nickel (Ni), arsenic (As), mercury (Hg), copper (Cu), cadmium (Cd), and chromium (Cr)) in soils may be intense industrialization, atmospheric transport, manure of farm animals, sewage sludge, and broad usage of the so-called synthetic fertilizers (Munir et al., 2023; Yavaş et al., 2022; Chatterjee and Abraham, 2022). The mechanisms by which toxic metals cause oxidative stress and damage the DNA are through inhibitation of photosynthesis, malfunction of enzymes, and imbalance of nutrients. The serious threat to the food chain and human health is the bioaccumulation of heavy metals, and therefore, efficient mitigation measures are outlined to this end (Soni et al., 2024; Chowardhara et al., 2024).

The third theme concerns Molecular and Genotypic Responses to Nanoparticle Exposure, appearing in 30 out of 49 papers. Studies show that there is genotypic variability in NP-induced responses, and that plant defence mechanisms are modulated by miRNAs, phytohormones, and epigenetic modifications (Munir et al., 2023; Basak et al., 2023; Ucar et al., 2024; Tripathi et al., 2024; Kumar et al., 2024). Integration of multi-omics is suggested to explore regulatory networks and biomarkers for heavy metal tolerance and nanoparticle interaction (Faizan et al., 2023a; Upadhyay et al., 2024).

Limitations of Current Mitigation Strategies and Research Gaps is the fourth theme, found in 28 out of 49 papers. Among their drawbacks are the costs, effectiveness, and environmental consequences of traditional methods like genetic engineering, soil improvement, and phytoremediation. Although there is encouraging physiological evidence supporting the advantages of NP, our comprehension of genotypic diversity and the molecular processes that contribute to NP-induced tolerance remains insufficient (Rai et al., 2023; Ghorbani et al., 2024; Yavaş et al., 2022; Chowardhara et al., 2024).

The fifth theme is Nano-Priming and Seed Treatment for Enhanced Stress Tolerance, which appears in 14 out of 49 papers. Nano-priming, i.e. the pretreatment of seed with nanoparticle is a promising technique of crop seed pre-treatment and could be used to enhance their germination, vigour, antioxidant responses, and stress tolerance under abiotic stress conditions and heavy metal stress. This alters physiological and molecular processes thereby enhances growth and production (Lee and Kasote, 2024; Yavari et al., 2023).

Silicon Nanoparticles in Heavy Metal Stress Alleviation represents the sixth theme, appearing in 13 out of 49 papers. Silicon nanoparticles (SiNPs) have been shown to counteract the effects of heavy metal toxicity by applying a multitude of mechanisms (e.g. by complexing with metals, reducing their absorption, increasing the activity of antioxidant enzymes, and modifying hormonal signaling). Studies show that more needs to be done in regards to further research in order to better comprehend molecular processes and make better use of SiNPs, which helps in growth and photosynthesis under adverse conditions (Singh et al., 2024; Asgher et al., 2024; Grewal et al., 2024; Aqeel et al., 2023).

The seventh theme concerns Nanoparticle-Mediated Redox and Antioxidant Regulation, found in 12 out of 49 papers. Nanoparticle use helps plants adapt to heavy metals and other environmental stress factors by regulating the synthesis and removal of reactive oxygen species (ROS) within hormetic ranges and the balance between pro- and antioxidants. This two-fold action assists the plants to adjust to stress by stimulating signaling pathways and act as antioxidant enzymes (Venzhik and Deryabin, 2023; Zhao et al., 2022; Bhattacharya et al., 2023; Kumari et al., 2024).

Nanozymes and Enzyme-Mimetic Nanoparticles for Heavy Metal Detoxification is the eighth theme, appearing in 8 out of 49 papers. Fresh solutions to eradicating reactive oxygen species and reducing the deleterious impact of heavy metals in plants can be created using enzyme-imitation nanoparticles, referred to as nanozymes. The techniques of application and their physicochemical properties influence their efficacies in providing specific ROS detoxification and overall immunity effects (Wang et al., 2025).

Environmental and Safety Considerations of Nanoparticle Use is addressed in 8 out of 49 papers. Despite the persistent concerns related to the environmental toxicity of nanoparticle and their bioaccumulation, as well as potential impact on the non-target organism, they potentially can mitigate heavy metal stress. Research indicates that rigorous control, assessment of the risks, and dosage optimization are the necessities in order to practice agriculture safely and sustainably (Yadav et al., 2024; Roy et al., 2024; Ghosh and Sarkar, 2022; Upadhyay et al., 2024).

### 3.4 Chronological review of literature

**Figure 3:**
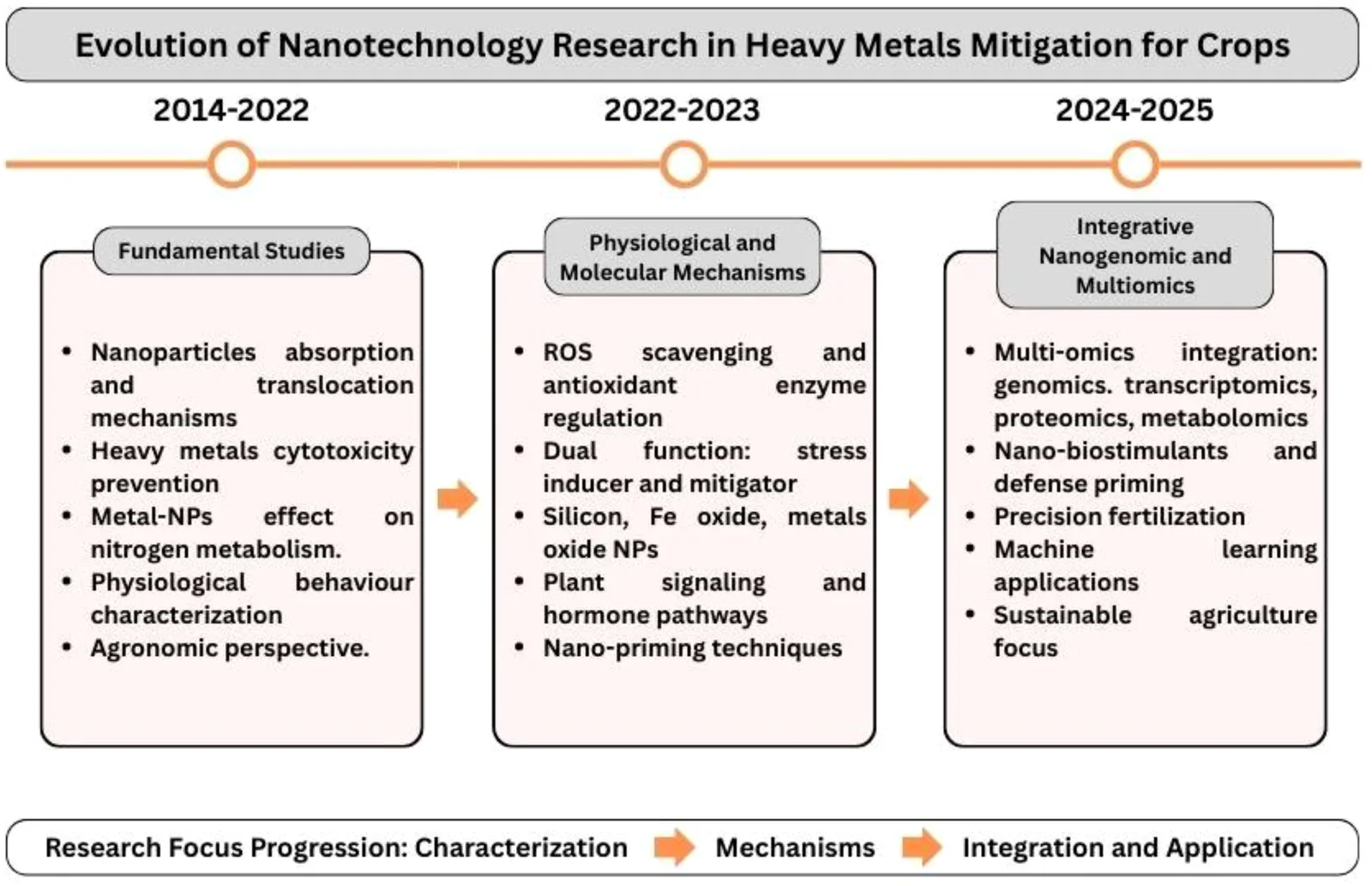
Research evolution of nanotechnology for heavy metals mitigation (2014-2025).

The development of research on the impact of heavy metal on crops with the use of nanotechnology has been achieved significantly over the last decade (**Figure 3**). The principal goals of the first research were to ensure better insight into the physiological and biochemical outcomes of heavy metal poisoning and study the nanoparticle as the possible mean of the mitigation. The studies that are described in the present section revolved around the genetic changes that were induced by nanoparticles and entailed in-depth molecular, proteomic and transcriptomic analyses. The new changes have focused on integrative nano-genomic options, which use multi-omics technology as a means of developing sustainable and precision agriculture options to enhance the resilience of crops and guarantee food safety.

The research evolution can be traced through three distinct periods. During the 2014-2020 period, foundational studies on nanoparticles in crop stress mitigation were established. Besides early studies highlighting the nanoparticle absorption, translocation and prevention of heavy metal cytotoxicity in crops, early investigations of the effects of metal nanoparticles on nitrogen metabolism and response of plants to the heavy metal toxicity became a matter of focus. These studies lay the foundation by characterizing the behavior of nanoparticles, their impact in physiology and their prospect in agronomy.

The 2022-2023 period marked an expansion into molecular and physiological mechanisms. Using nanoparticles, research focused on the molecular underpinnings of abiotic stress tolerance, with particular attention to ROS scavenging, antioxidant enzyme regulation, and the dual function of nanoparticles as stress inducers and mitigators. The use of particular nanoparticles, such as silicon, iron oxide, and metal oxides, to reduce heavy metal toxicity, nanomaterial interactions with plant signaling and hormone pathways, and nano-priming techniques were all investigated, with an increasing emphasis on transcriptomic and proteomic responses.

Most recently, the 2024-2025 period has seen the development of integrative nano-genomic and multi-omics approaches. To clarify the genotypic modulation brought on by nanoparticles under heavy metal stress, recent research has focused on nano-genomic frameworks that integrate genomics, transcriptomic, metabolomics, and proteomics. Nanoparticle-mediated defense priming, nano-enabled agrochemicals, and nano-biostimulants that improve plant tolerance are all being investigated. In order to maximize the application of nanoparticles while addressing regulatory and environmental safety concerns, there is a great deal of focus on precision fertilization, sustainable agriculture, and machine learning.

### 3.5 Theoretical and practical implications

#### 3.5.1 Theoretical implications

Combined with the literature reviewed, the literature informs the theoretical position that nanoparticles (NPs) can alter plant tolerance to heavy metal (HM) stress by reprogramming and modifying the genes (genotypic) and molecules (molecular) as well as physiological changes. To explain NP-induced genotypic modulation, alterations in gene expression, metabolomic, and anti-oxidative defence pathways can support the new integration of multi-omics tools (Munir et al., 2023; Basak et al., 2023; Zou et al., 2022). The finding provides additional validation to the importance of genetic diversity in shaping NP effectiveness as diverse crop genotypes respond differently to NP applications under exposure to HM stress. The data supports the notion that genotype-specific biological events that can affect NP-induced tolerance to a substantial degree include miRNA control and epigenetic changes (Ucar et al., 2024; Basak et al., 2023; Faizan et al., 2023). A platform of delving into the complex interactions between nanoparticles and plant genomes, proteomes and metabolomes is formed with the combination of nanotechnology and omics approaches. Such fusion method allows to discover sensitive biomarkers and determine mechanistic processes, thereby deepening our knowledge on the basic plant stress biology and nanobiotechnology (Munir et al., 2023; Upadhyay et al., 2024; Chowardhara et al., 2024).

The analysis of the data demonstrates the importance of the combination of molecular and genotypic features, which refutes the previous mitigation strategies that focus on physiological consequences mostly. Such a shift is in line with contemporary ideas in systems biology and will enable the development of custom nanotechnology-based solutions to suit specific genotypes of crops and custom culture conditions (Ningombam et al., 2024; Francis et al., 2024; Jiang et al., 2023).

#### 3.5.2 Practical implications

Nanoparticles (NPs) are proven to minimize both absorption and accumulation of heavy metals (HM) in plants besides enhancing growth and yield characteristics. This discovery has implications in the development of nano-fortified agrochemicals and amendments in polluted soils to enhance food security and agriculture yields and produce (Chen et al., 2024; Ghorbani et al., 2024; Iqbal et al., 2024). The nano-genomic methodology that forms the use of transcriptomic, proteomics, and metabolomics will enable the generation of pollution-safe cultivars and precision agricultural uses through offering a practical way of breeding and designing crop variants that are better in their NP responsiveness and HM tolerance levels (Munir et al., 2023; Lee and Kasote, 2024; Faizan et al., 2023b).

Given that the effects of NPs may vary across crop genotypes and conditions and that NP formulations, doses, and delivery methodologies vary in both effectiveness and potential phytotoxicity or ecological hazard, genotype-specific optimization of formulations and doses and delivery strategies is necessary (Yavari et al., 2023; Roy et al., 2024; Upadhyaya and Roychoudhury, 2024). Combinations of NPs and existing biotechnologies, such as CRISPR gene editing and nano-priming, offer scalable methods of increasing crop resilience. The practices of the industry that work to attain climate-smart agriculture and sustainable intensification can be subjected to these measures (Rai et al., 2023; Lee and Kasote, 2024; Yavari et al., 2023).

In order to ensure responsible deployment in agricultural systems, regulatory frameworks and safety assessments must change to address the dual nature of NPs, balancing their advantageous roles in stress mitigation against potential cytotoxic and genotoxic effects (Yadav et al., 2024; Kumar et al., 2024; Ahmad et al., 2023). Policymakers and agronomists can use the encouraging findings from meta-analyses and machine learning models that pinpoint important soil and NP properties that affect HM bioavailability and plant uptake to inform NP application strategies that maximise environmental and human health outcomes (Chen et al., 2024; Chowardhara et al., 2024; Zulfiqar et al., 2024).

### 3.6 Gaps and future research directions

The reviewed literature has critically identified significant research gaps in areas such as limited analysis of genotypic diversity with NPs applications, inadequate multi-omics integrations, deficiency of long-term and field-scale confirmation, undecided molecular mechanisms of NP-genotype relations, optimization of nanoparticle preparations and doses, unsatisfactory understanding of epigenetic and transgenerational effects, environmental and eco-toxicological threats of nanoparticles, and incomplete research on seed nano-priming effects on genotypic modulation which are discussed in detail in **Table 2**.

**Table 2:** Key research gaps and proposed future directions for nano-genomic approaches in enhancing crop tolerance to heavy metal stress.

Gap area	Description	Future research directions	Justification	Research priority
Limited Genotypic Diversity Analysis in NP Studies	Most studies focus on physiological responses to nanoparticles under heavy metal stress but lack comprehensive analysis of genotypic diversity and variability across crop species.	Conduct large-scale comparative genomic and epigenetic studies across diverse crop genotypes to elucidate genotype-specific nanoparticle tolerance mechanisms. Integrate multi- omics data to identify genetic markers linked to NP-induced tolerance.	Understanding genotypic variability is critical for breeding and engineering crops with enhanced NP-mediated heavy metal tolerance (Munir et al., 2023; Basak et al., 2023; Faizan et al., 2023a).	High
Incomplete Integration of Multi- Omics Data	Current omics studies often analyze transcriptomic, proteomics, or metabolomics separately, with limited integration and standardization across datasets.	Develop standardized protocols and bioinformatics pipelines for integrative multi- omics analyses (transcriptomic, proteomics, metabolomics, epigenetics) to capture holistic NP-induced molecular responses.	Integrated omics approaches provide systems-level insights necessary for deciphering complex NP-plant interactions and genotypic modulation (Munir et al., 2023; Wang et al., 2025; Chowardhara et al., 2024).	High
Lack of Long-Term and Field-Scale Validation	Most research is conducted under controlled laboratory or greenhouse conditions with short-term exposure, lacking field trials and long-term environmental impact assessments.	Design and implement multi- season field experiments to evaluate the efficacy, safety, and environmental fate of nanoparticles in diverse agroecosystems under realistic heavy metal contamination scenarios.	Field validation is essential to translate laboratory findings into practical, scalable agricultural applications and assess ecological risks (Tripathi et al., 2024; Kumari et al., 2023; Ahmad et al., 2023).	High
Unclear Molecular Mechanisms of NP- Genotype Interactions	The precise molecular pathways and gene networks modulated by nanoparticles in different genotypes remain poorly characterized.	Employ CRISPR/Cas gene editing and functional genomics to dissect key genes and regulatory networks involved in NP-induced heavy metal tolerance across genotypes.	Mechanistic understanding enables targeted crop improvement and rational design of nano- enabled agrochemicals (Rai et al., 2023; Ucar et al., 2024; Faizan et al., 2023b).	High
Optimization of Nanoparticle Formulations and Dosages	There is no consensus on optimal nanoparticle types, sizes, coatings, and application dosages for maximizing efficacy while minimizing toxicity.	Systematically evaluate physicochemical properties of nanoparticles and dose- response relationships in multiple crop species and genotypes to establish safe and effective application guidelines.	Optimized formulations are necessary to avoid phytotoxicity and environmental contamination while ensuring stress mitigation (Wang et al., 2025; Francis et al., 2024; Upadhyaya and Roychoudhury, 2024).	Medium
Insufficient Understanding of Epigenetic and Transgenerational Effects	Few studies address how nanoparticles influence epigenetic modifications and whether induced tolerance traits are heritable.	Investigate nanoparticle- induced epigenetic changes (DNA methylation, histone modifications, miRNA regulation) and their transgenerational inheritance in crops under heavy metal stress.	Epigenetic insights can reveal stable, heritable tolerance mechanisms and inform breeding strategies (Basak et al., 2023; Ningombam et al., 2024).	Medium
Environmental and Ecotoxicological Risks of Nanoparticles	Limited data exist on the long-term environmental fate, bioaccumulation, and non-target organism impacts of nanoparticles used in agriculture.	Conduct comprehensive ecotoxicological assessments, including soil microbiome studies, trophic transfer analyses, and nanoparticle degradation pathways in field conditions.	Responsible use of nanoparticles requires understanding and mitigating potential ecological risks (Kumari et al., 2023; Ahmad et al., 2023; Ghosh and Sarkar, 2022).	High
Limited Application of Machine Learning for Predictive Nano-genomics	Although some studies use machine learning to analyze omics data, predictive modeling of genotype-specific NP responses is underdeveloped.	Develop machine learning models integrating multi-omics and phenotypic data to predict crop genotype responses to nanoparticle treatments under heavy metal stress.	Predictive tools can accelerate screening and selection of tolerant genotypes and optimize NP applications (Chowardhara et al., 2024; Upadhyay et al., 2024).	Medium
Underexplored Role of Phytohormone- Nanoparticle Crosstalk	The interaction between nanoparticles and plant hormonal signaling pathways in modulating heavy metal tolerance is not fully elucidated.	Investigate molecular crosstalk between nanoparticles and phytohormones (e.g., auxin, salicylic acid, melatonin) using omics and hormone quantification in diverse genotypes.	Hormonal regulation is central to stress responses; understanding NP influence can enhance tolerance strategies (Rai et al., 2023; Kumar et al., 2024).	Medium
Limited Research on Seed Nanopriming Effects on Genotypic Modulation	Seed nanopriming shows promise in enhancing stress tolerance, but its effects on genotypic modulation and underlying molecular mechanisms remain unclear.	Perform genome-wide and epigenome-wide analyses of seed nanoprimed plants under heavy metal stress to identify induced genetic and epigenetic changes across genotypes.	Elucidating seed priming effects at the genotypic level can improve early- stage stress resilience and crop establishment (Lee and Kasote, 2024; Yavari et al., 2023).	Medium

## 4 Conclusion

The suggested nano-genomic approach provides a thorough framework for analyzing and utilizing genotype-specific nanoparticle responses for sustainable agriculture. It does this by utilizing multi-omics tools in conjunction with sophisticated bioinformatics and machine learning. There are still many unanswered questions about long-term environmental effects, nanoparticle accumulation, ecotoxicology, and regulatory considerations, despite the fact that nanoparticles offer advantages over traditional techniques by offering targeted and comprehensive mitigation of heavy metal toxicity. To ensure the safe, efficient, and scalable use of nanoparticles, it is essential to conduct field-scale validations and conduct interdisciplinary research that integrates environmental science, genomics, agronomy, and nanotechnology. In the face of rising heavy metal contamination globally, developing nano-genomic techniques has the potential to create resilient crop cultivars, protect soil and food safety, and promote sustainable agricultural practices.

## Data Availability

The original contributions presented in the study are included in the article/supplementary material. Further inquiries can be directed to the corresponding author.
