# Correction: Genotypic modulation of nanoparticles-induced tolerance in heavy metals-stressed crops; a literature review

**DOI:** 10.3389/fpls.2026.1984719

**Published:** 2026-09-15

**Authors:** Muhammad Hammad, Aasma Riaz, Laraib Chouhdary, Muhammad Kabir, Patricio R. De los Rios-Escalante

**Affiliations:** 1 Department of Biological Sciences, Thal University Bhakkar, Bhakkar, Punjab, Pakistan; 2 Department of Botany, University of Agriculture, Faisalabad, Punjab, Pakistan; 3 Departamento de Ciencias Biologicas y Químicas, Facultad de Recursos Naturales, Universidad Catolica de Temuco, Temuco, Chile

**Keywords:** epigenetic regulations, genotypic modulation, heavy metals stress, nano-genomics, nanoparticles-induced tolerance

“Chatterjee and Abraham, 2022” was incorrectly cited in the article as “Chatterjee & others”. The citation has now been updated in section **3 Results**, subsection *3.3 Thematic review of literature*, paragraph 3 and should read:

“The factors that can contribute to the presence of heavy metals (zinc (Zn), lead (Pb), nickel (Ni), arsenic (As), mercury (Hg), copper (Cu), cadmium (Cd), and chromium (Cr)) in soils may be intense industrialization, atmospheric transport, manure of farm animals, sewage sludge, and broad usage of the so-called synthetic fertilizers (Munir et al., 2023; Yavaş et al., 2022; Chatterjee and Abraham, 2022)”.

“. Ia (hernia), Il (colon) or Yc (Pancreas)” has been removed from section **3 Results**, subsection *3.5.2 Practical implications*, paragraph 2. The correct text reads as follows:

“Given that the effects of NPs may vary across crop genotypes and conditions and that NP formulations, doses, and delivery methodologies vary in both effectiveness and potential phytotoxicity or ecological hazard, genotype-specific optimization of formulations and doses and delivery strategies is necessary (Yavari et al., 2023; Roy et al., 2024; Upadhyaya and Roychoudhury, 2024)”.

A round bracket was used instead of a semicolon in “(Upadhyay et al., 2024) Jamil et al., 2023)”. A correction has been made in section **1 Introduction**, paragraph 3:

“The conceptual framework of this review is based on the connection between genotypic diversity of plants, their tolerance against heavy metals and mitigation by application of nanotechnology (Upadhyay et al., 2024; Jamil et al., 2023)”.

The original version of this article has been updated.

